# Supplementary material for: A Global Screen for Assembly State Changes of the Mitotic Proteome by SEC-SWATH-MS
Source: Cell Syst. 2020 Feb 26;10(2):133–155.e6. doi: 10.1016/j.cels.2020.01.001 (PMC7042714; doi:10.1016/j.cels.2020.01.001)

Q13443 | ADAM9_HUMAN | ADAM9 KIAA0021 MCMP MDC9 MLTNG
Monomer MW [kDa]: 90.556 Monomer expected elution fraction: 41

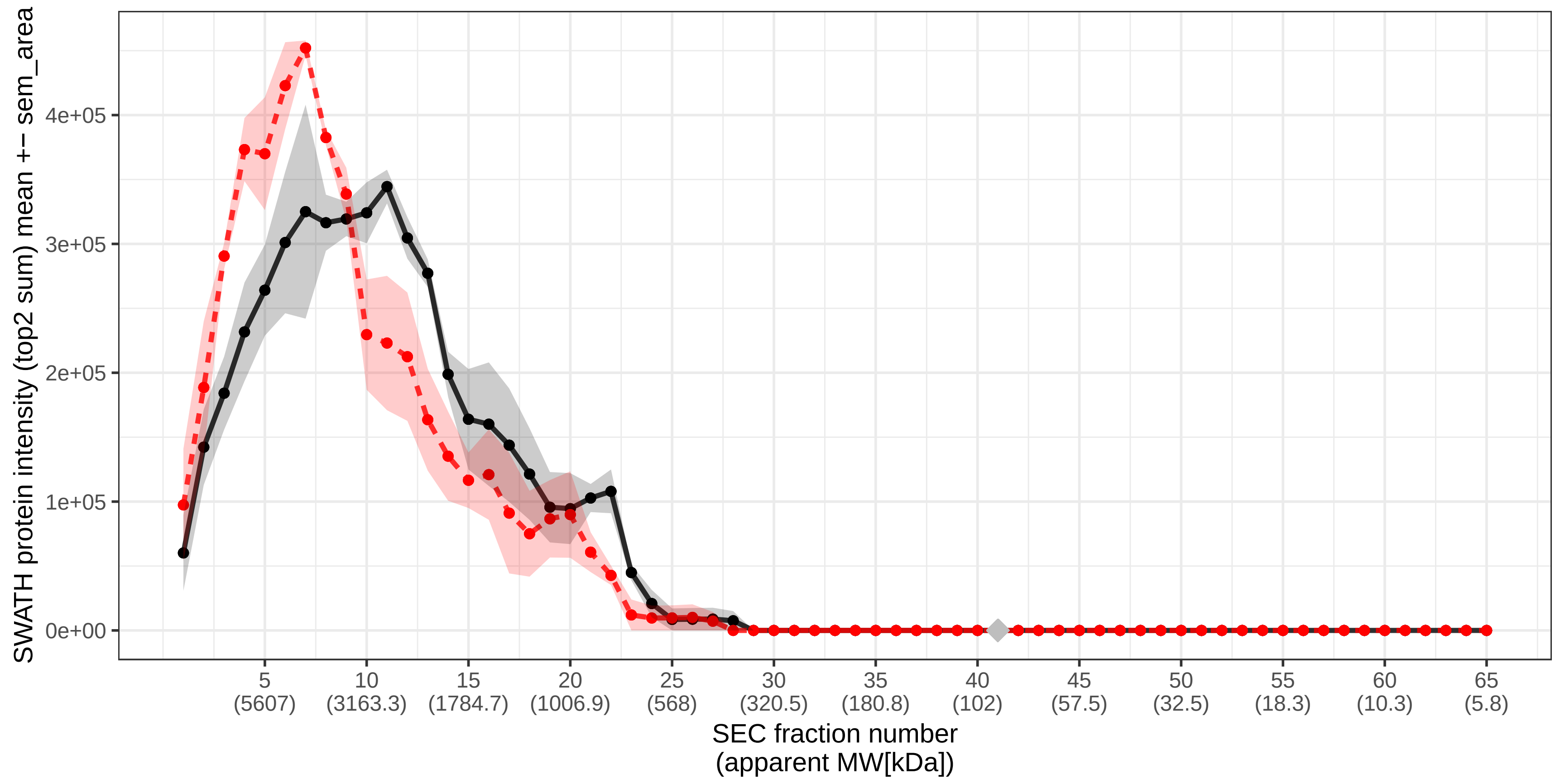

Supplement: Data S1. SEC-SWATH-MS Protein Chromatograms, Related to Figure 1 [file mmc6.zip › SECchrom_Q13443_ADAM9_HUMAN_ADAM9_KIAA0021_MCMP_MDC9_MLTNG.pdf]
